# Cancer-Associated Thrombotic Microangiopathy: Literature Review and Report of Five Cases

**DOI:** 10.3390/life14070865

**Published:** 2024-07-10

**Authors:** L. Posado-Domínguez, A.-J. Chamorro, E. Del Barco-Morillo, M. Martín-Galache, D. Bueno-Sacristán, E. Fonseca-Sánchez, A. Olivares-Hernández

**Affiliations:** 1 Medical Oncology Department, University Hospital of Salamanca, 37007 Salamanca, Spain; 2 Biomedical Institute Research of Salamanca (IBSAL), 37007 Salamanca, Spain; 3 Internal Medicine Department, University Hospital of Salamanca, 37007 Salamanca, Spain; 4 Faculty of Medicine, University of Salamanca, 37008 Salamanca, Spain; 5 Pediatrics Department, Pediatrics Oncology Section, University Hospital of Salamanca, 37007 Salamanca, Spain; 6 Anatomical Pathology Department, University Hospital of Salamanca, 37007 Salamanca, Spain

**Keywords:** cancer, thrombotic microangiopathy, pulmonary hypertension, chemotherapy, adenocarcinoma, healthcare management

## Abstract

Thrombotic microangiopathy (TMA) is an anatomopathological lesion mediated by endothelial dysfunction and characterized by the creation of microthrombi in small vessels. In patients with cancer, it may be due to toxicity secondary to chemotherapy, tumor embolization, or hematopoietic progenitor transplantation. Cancer-associated TMA is an underestimated entity that generally appears in the final stages of the disease, although it may also be the initial manifestation of an underlying cancer. Support treatment is necessary in all cases and, depending on the cause, different targeted therapies may be used. The prognosis is very poor. In this article we present a comprehensive review of the existing literature on the physiological mechanisms of cancer-associated TMA. Afterwards, five clinical cases will be presented of patients who developed TMA and were diagnosed in our Department in 2023. We present a discussion of the different causes that triggered the condition, the possible reasons behind the underestimation of this pathology, and the measures that may be adopted.

## 1. Introduction

### 1.1. Background

Thrombotic microangiopathy (TMA) is an anatomopathological lesion mediated by endothelial dysfunction and characterized by the formation of microthrombi in small vessels [1]. This leads to microangiopathic hemolytic anemia (MAHA) as a result of a mechanical injury when red blood cells move through the damaged capillaries, thrombocytopenia (<150,000 platelets/mL or a decrease by 30% in the number of platelets compared to basal values), and ischemic lesions in target organs secondary to the direct damage of microthrombi in vessels and the decreased supply of oxygen and nutrients [2]. Since the year 2005, attempts have been made at establishing different diagnostic criteria for TMA; two of the best classifications available are those by the Blood and Marrow Transplant Clinical Trials Network (BMT-CTN) and the International Working Group (IWG), and they include variables such as schistocytes per field, level of lactate dehydrogenase (LDH), decrease in haptoglobin, or negative direct and indirect Coombs test [3,4].

The onset of the symptoms may be triggered by different causes, including an exaggerated response of the organism to infections caused by different viruses or bacteria. This response is mediated by the activation of the alternative complement pathway and is part of what is known as hemolytic uremic syndrome (HUS) [5]. The complement may be involved in some cases of TMA associated with active cancer, either as part of a paraneoplastic syndrome or secondary to the administration of chemotherapy (CT) such as gemcitabine or oxaliplatin [6]. Sometimes, the initial mechanism is unknown, as in the case of thrombotic thrombocytopenic purpura (TTP), in which the metalloenzyme ADAMTS13, a protease that transforms von Willebrand factor (FVW), which has a powerful pro-coagulant activity, into low-molecular-weight polymers (with low pro-coagulant activity), decreases its function, and promotes the formation of platelet aggregates. There are cases of ADAMTS13 deficiency with congenital origin, although in most cases the cause is the development of autoantibodies against the metalloenzyme [7,8]. ADAMTS13 deficiency may be observed in some cases of cancer-associated TMA [9]. Poorly controlled arterial hypertension causes endothelial damage via the intimal proliferation and release of pro-coagulant substances; this would be the mechanism of some rheumatologic diseases such as scleroderma. Intimal proliferation is the initial mechanism of some cases of TMA associated with tumor microembolism and TMA secondary to kidney damage induced by chemotherapy or radiation [7]. Some subtypes of active cancer may cause an excessive production of thrombin, which triggers platelet consumption and thrombotic phenomena. These symptoms, known as disseminated intravascular coagulation (DIC) may ultimately trigger TMA [8,10]. Different causes of TMA are included in Table 1.

### 1.2. Objectives

Cancer-associated TMA is a very broad topic; however, there are few references in the literature that apply theoretical findings to complex clinical cases that make it possible to understand the process behind this condition and the general evolution of the patients. Through this comprehensive literature review, we have attempted to synthesize as much as possible five cancer-associated TMA cases that were registered in our Department during the year 2023.

### 1.3. Rationale and Knowledge Gap

We may consider that there is a subgroup of TMA which is associated with active cancer. In turn, cancer-associated TMA may be due to three causes: (1) associated with chemotherapy; (2) associated with active cancer; and (3) associated with hematopoietic stem cell transplantation [2,6,7] (Table 2). This study will discuss the first two causes, because the third one is increasingly less common in medical oncology, while it is more frequently discussed in hematology.

Cancer-associated thrombosis has been documented since the 1960s [11]. The first reports date back to clinical cases of adenocarcinomas with a predominance of those with gastric origin and mucinous characteristics [12]. From an anatomopathological perspective, it reveals images of endoluminal thrombi, fibrinoid necrosis of arterioles, and intimal thickening. Kidney biopsies have shown thickening of glomerular capillaries, fibrin thrombi, necrotic endothelial cells, and complement C3 deposition [13]. They also have shown changes in the morphology of arterioles and lung vessels with intimal and medial hypertrophy, intimal fibrosis, and increased alveolar macrophages [14]. Many of these findings are observed in autopsies, since living patients are rarely biopsied during the diagnostic process. Some series of post-mortem studies show a prevalence of TMA of 1–3% in autopsies of oncological patients, which suggests that TMA could be underdiagnosed [14,15].

One typical finding in TMA is the presence of schistocytes in a smear due to the fragmentation of red blood cells, but their absence does not preclude the diagnosis [4]. From an analytical perspective, there may be increased levels of indirect bilirubin (Br), LDH, and reticulocytes, together with a decrease in haptoglobin and platelets [6,13]. Creatinine (Cr) may increase as a result of decreased glomerular filtration. From a coagulation standpoint, there is a decrease in prothrombin time (PT) and in the concentration of fibrinogen, together with an increase in D-dimer levels [6,7,13]. In some cases, coagulation may be normal or show slight alterations [13]. The urine analysis may reveal nephrotic-range proteinuria (>3 g/24 h), hematuria, and granular and hyaline casts [1,2].

Clinically, symptoms are observed at different levels (Table 3). At the kidney level, there is oligoanuria, limb and periorbital edema, intense asthenia, nausea, and vomiting. The appearance of acute kidney insufficiency (AKI) may cause hyperkalemia, metabolic acidosis, and confusion [16]. Renal hypoperfusion secondary to kidney damage releases renin, which activates the renin–angiotensin–aldosterone system and increases arterial tension [5,6]. Platelet consumption may involve skin lesions and bleeding at different levels (hematuria, melena, metrorrhagia). There may also be fever [2]. When the endothelial dysfunction affects the lung vessels in TMA, this is known as pulmonary tumor thrombotic microangiopathy (PTTM) [17]; the symptoms are unspecific, and they may evolve throughout weeks or months, including progressive mild dyspnea that becomes disabling, chest pain, and coughing. The physical examination may reveal tachycardia and tachypnea, and the electrocardiogram (ECG) may show signs of right ventricular volume overload with S1Q3T3 pattern [15]. At the pulmonary level, CT such as mitomycin [7] or tumor microemboli [14] may, in very rare cases, trigger pulmonary hypertension (PH) which progresses rapidly. Most cases are associated with lung or breast tumors, or with gastric carcinomas. They may appear as severe hypoxemia, with computerized tomography scans that do not generally show thrombi, whereas radionucleotide ventilation/perfusion (V/Q) scans generally show multiple subsegmental perfusion defects with normal ventilation. Prognosis is fatal [18]. The gastrointestinal tract may also be affected. The obstruction of intestinal microvessels causes ischemia which starts with very intense abdominal pain [19]. The central nervous system may also be affected. From a clinical perspective, there may be decreased consciousness, blindness, aphasia, and loss of strength and sensitivity [20].

- Chemotherapy-associated TMA

The drugs most commonly associated with CT-associated TMA are platinum derivatives, mitomycin C, gemcitabine, bevacizumab, and tyrosine kinase inhibitors (TKIs) such as regorafenib or sunitinib [9,21].

Platinum derivatives are nephrotoxic. Direct damage on the renal endothelium releases coagulation-promoting substances [22]. Cisplatin increases platelet reactivity and releases phospholipase A2 [23]. Oxaliplatin has been associated with the release of antibodies against red blood cells and platelets [23,24]. Platinum-associated TMA is a severe and potentially lethal complication. Administration of the drug must be suspended and support treatment with control of symptoms must be initiated. Plasmapheresis and anticomplement therapies such as eculizumab or cytokine regulation with the anti-CD20 monoclonal antibody rituximab have not proven effective [23,24].

Gemcitabine has been associated with TMA secondary to accumulated toxicity after 6–8 months of treatment [25]. The incidence of thrombotic complications is low (below 0.5%) [13], and one of the causes could be an acute lesion of the renal endothelium that releases pro-coagulant substances and proinflammatory cytokines that trigger the coagulation cascade and the creation of microthrombi. In some cases, patients have received rituximab and eculizumab, a humanized monoclonal antibody that binds to the complement C5 protein, with promising results, although evidence is still limited [26]. Although it may cause kidney damage, the main presentation of mitomycin C-associated TMA is the appearance of acute respiratory insufficiency associated with respiratory distress. This is induced by the damage on the pulmonary endothelium with the creation of microthrombi and the increased concentration of macrophages in the lung microvessels. It appears 1–2 months after the last dose [13,27]. These patients show high levels of plasma thrombomodulin and plasminogen activation, and these alterations are similar to those found in HUS [28]. Eculizumab and plasmapheresis have proven effective in some cases [29]. Bevacizumab is a monoclonal antibody that inhibits the activity of vascular endothelial growth factor (VEGF-A), a protein that promotes angiogenesis [21]. The development of bevacizumab-associated TMA is based on the endothelial damage it causes to renal vessels and the deregulation of the complement system. Eculizumab has been effective in some cases [30]. There are also described cases of TMA associated with TKIs, including regorafenib and sunitinib. These drugs cause acute glomerular renal damage and AHT, proteinuria, and TMA [31]. The complement system does not seem to be affected. Eculizumab has not been effective in cases of TKI-associated TMA [32].

- Cancer-associated TMA

This is mainly associated with gastric, breast, and lung cancer, and with cancer of unknown origin [6,7]. The main etiology is adenocarcinoma. One of the obstacles for diagnosis is the need for clinical suspicion, because this may appear both in the final stages of the metastatic disease and in the initial stages, as the first manifestation of an underlying cancer [33,34]. Tumor cells trigger the coagulation cascade via the tissue factor (TF) and the induction of proinflammatory cytokines, such as tumor necrosis factor alpha (TNF-a), interleukin 1 (IL-1), interleukin 6 (IL-6), interleukin 8 (IL-8), and transforming growth factor beta (TGF-b) [13]. TNF-a is a powerful inductor of neutrophil degranulation. It stimulates the production of chemotaxis molecules on the endothelium and it also stimulates the coagulation cascade through the activation of the intrinsic pathway, which induces the expression of TF on the leukocyte surface, the negative regulation of natural anticoagulants (via protein C and heparin-antithrombin), and the release of phospholipase A2 (PLA2), which improves the function of the platelets and, together with IL-6, promotes the formation of thrombin [35,36]. IL-1 triggers the release of mediators such as IL-6 and IL-8. IL-6, which is secreted by the liver and the leukocytes, is involved in the acute stage of inflammation, and triggers a proinflammatory cascade via the activation of the JAK-STAT3 pathway [37]. It also stimulates the liver production of fibrinogen and C-reactive protein (CRP) [37]. CRP has prothrombotic activity via the activation of the extrinsic pathway of coagulation: it induces the expression of TF on endothelial cells and monocytes [38]. IL-8 is proinflammatory, and it is produced by macrophages, epithelial cells, smooth muscle cells of the respiratory pathways, and endothelial cells [39]. Some studies have observed the presence of IL-8 receptors in platelets, which would trigger a pro-coagulant action [40]. TGF-b stimulates the production of fibroblasts by affecting the development of fibrosis in chronic inflammatory processes such as cancer [41]. Some authors have mentioned that it could indirectly affect coagulation through the promotion of oxidative stress and the generation of oxygen reactive species, which could increase the expression of TF and proteases [37,42].

The deregulation of the complement is associated with cancer-induced TMA. The complement proteins C3a and C5a, whose expression is elevated in different tumors such as lung adenocarcinoma or ovarian carcinoma, participate in the formation of new tumor blood vessels, which induce the expression of VEGF and platelet-derived growth factor subunit A (PDGFA), which might cause pulmonary TMA [43,44]. C3a and C5a may induce HUS in patients with cancer and a severe infection [45]. When C5a binds to thrombin, it triggers the release of IL-6, IL-8, and TNF, which initiates a prothrombotic and proinflammatory cascade [46].

In summary, interleukins play an essential role in the development of cancer-associated TMA by inducing a proinflammatory state. Il-1, Il-6, and IL-8 may act directly on the structure of platelets and red blood cells and alter the coagulation parameters, which causes the hyperactivation of platelets and may alter the structure of red blood cells to trigger the creation of thrombi [41]. This process is vital in some tumors such as pancreatic adenocarcinoma or gastric adenocarcinoma, in which some meta-analyses mention the possibility to initiate prophylactic anticoagulation after diagnosis [47].

## 2. Case Reports

We present five clinical cases of patients diagnosed with cancer-associated TMA who were treated by the medical oncology department of the University Hospital of Salamanca, in the region of Castile and León, Spain, in the year 2023. The clinical and analytical data are summarized in Table 4. Each case is divided into two parts, a short oncological history to know the oncological evolution, and the reasons for consultation and diagnostic conclusion of the case (Table 4).

### 2.1. Case 1

#### 2.1.1. Oncological History

The patient is a 59-year-old woman without a relevant medical history. She was diagnosed in April 2021 with multifocal infiltrating lobular carcinoma (ILC) of the breast, HER2 positive, OR 80%, PR 80%, Ki-67 10%, and ypT1 N1a.

She received neoadjuvant therapy (epirubicin and cyclophosphamide followed by paclitaxel-trastuzumab-pertuzumab) and underwent mastectomy and lymphadenectomy with adjuvant radiotherapy and CT (trastuzumab-emtansine-atezolizumab). Then, 18 months later, she presented bone and liver recurrence. The first line of treatment of the metastatic disease was started with trastuzumab-deruxtecan, which she was still receiving when she was assessed.

#### 2.1.2. Reasons for Consultation

She was admitted in December 2023 by the medical oncology department with asthenia and choluria of several days of evolution. The analysis showed pancytopenia (platelets 39 × 10^3^/μL, leukocytes 1.52 × 10^3^/μL, hemoglobin 10.9 × 10^3^/μL), renal function deterioration (GF CKD-EPI 20 mL/min/1.73 m^2^), alteration in the hepatic profile (bilirubin -Br- 2.9 mg/dL, direct Br 1.9 mg/dL, ALT 105 U/L, AST 82 U/L, GGT 121 U/L), elevated LDH (455 U/L), and decreased fibrinogen (103.1 mg/dL).

A swab was performed, and it revealed the presence of over 5% of schistocytes in the peripheral blood.

During admission, the oncological involvement was assessed with an abdominal, pelvic, and thoracic CT scan that revealed the progression of the disease on the liver and the axial skeleton.

Based on the clinical and analytical findings and on the peripheral blood swab, the diagnosis was TMA secondary to tumor infiltration of the bone marrow caused by bone metastasis, and hepatopathy secondary to liver metastasis. Treatment was initiated with weekly cisplatin, and three cycles were administered during admission with good initial response. There was progressive clinical and analytical improvement, and the patient was discharged 23 days later to start ambulatory treatment. She consulted again 10 days after discharge with fever and minimal effort dyspnea. Support measures were implemented. The analysis revealed hypoxemia (pO_2_ 50 mmHg), deterioration of the liver and kidney function, and signs of myocardial damage (elevated troponin T and proBNP). An emergency swab showed an increase in the number of circulating schistocytes.

The patient died in the emergency room three hours after the start of the intervention. The case was diagnosed as TMA secondary to cancer with indirect data compatible with PTTM (hypoxemia and increase in troponin T and proBNP).

### 2.2. Case 2

#### 2.2.1. Oncological History

The patient is a 78-year-old woman who was diagnosed in October 2022 with gastric adenocarcinoma pT3 N3b (32 positive lymph nodes out of 57 isolated nodes) M0, who underwent laparoscopic lymphadenectomy D2 and subtotal gastrectomy. She received adjuvant therapy with 6 cycles of capecitabin and oxaliplatin (CAPOX), which ended in May 2023. One month after the treatment she started showing weight loss, anorexia, and asthenia. The analysis showed elevated levels of tumor marker CA 72.4. The abdominal, pelvic, and thoracic CT scan and bone scintigraphy revealed bone metastasis (right femur) and peritoneal carcinomatosis. The first line of treatment for the metastatic disease was initiated with paclitaxel.

#### 2.2.2. Reasons for Consultation

She was admitted as an emergency in July 2023 with deterioration of her general condition, moderate effort dyspnea, anorexia, asthenia, and poor pain control on the lumbosacral area. She had a basal O_2_ saturation of 87%. The analysis revealed alterations in coagulation (D-dimer 35.1 mg/L, fibrinogen 116.4 mg/dL), pancytopenia (platelets 24 × 10^3^/μL, leukocytes 2.96 × 10^3^/μL, hemoglobin 8.9 g/dL), and elevated LDH (1657 U/L). An angioCT scan of the pulmonary arteries was conducted and it did not show evidence of pulmonary embolism (PE), although the pulmonary arteries were dilated. The ECG showed a deviation of the electrical axis to the right, right bundle branch block, and tall and broad P waves in V1–V3, compatible with pulmonary hypertension (PH). A swab revealed the presence of circulating schistocytes and erythroblasts. The clinical diagnosis was DIC and TMA secondary to tumor infiltration to the bone marrow; and indirect data were compatible with PTTM (low O_2_ saturation, dilation of pulmonary arteries, and ECG alterations compatible with PH).

Considering the poor performance status of the patient (ECOG 4, Karnofsky 20%), symptom control was decided. The patient died 14 days after her admission due to multiple organ failure in the context of acute respiratory insufficiency.

### 2.3. Case 3

#### 2.3.1. Oncological History

The patient is a 73-year-old woman who had been diagnosed in March 2016 with diffuse type gastric signet ring cell adenocarcinoma with discohesive pattern pT1b pN0, who had been operated with subtotal gastrectomy without adjuvant therapy.

#### 2.3.2. Reasons for Consultation

She was admitted as an emergency in July 2023 with moderate effort dyspnea of weeks of evolution and asthenia. The blood analysis showed pancytopenia (platelets 32 × 10^3^/μL, leukocytes 4.31 × 10^3^/μL, hemoglobin 8.8 g/dL), elevated D-dimer (20.3 mg/L), and decreased ADAMTS-13 activity (30%). The patient also presented a marked elevation in LDH (2793 U/L) and NT-proBNP (3243 pg/mL).

A thoracic X-ray was performed, as well as a CT scan of the pulmonary arteries, which revealed bilateral pleural effusion and dilation of right cavities associated with PH. No pulmonary embolism was observed. The abdominal, pelvic, and thoracic CT scan revealed multiple involvement of the axial skeleton and right pelvis. Pleural fluid was obtained via thoracocentesis, and it was compatible with metastasis of gastric adenocarcinoma.

During admission, a swab revealed circulating schistocytes and erythroblasts, which were compatible with secondary microangiopathic hemolytic anemia. The symptoms were classified as bone recurrence (axial skeleton, right pelvis) and bilateral pleural recurrence after gastric adenocarcinoma, TME secondary to active cancer with bone marrow invasion, and HP secondary to PTTM.

Given the diagnosis of TMA secondary to cancer, CT was administered with a regime of carboplatin and paclitaxel, together with support treatment (oxygen therapy, serum therapy). The patient showed a poor evolution and died 5 days after her admission as a consequence of multiorgan failure secondary to TMA and PTTM.

### 2.4. Case 4

#### 2.4.1. Oncological History

The patient is a 70-year-old woman with a history of IDC of the left breast pT1b pN0 M0 diagnosed in August 2019, which received adjuvant therapy with anastrozole. She had also been diagnosed in November 2021 with high-grade endometrial carcinosarcoma pT2 N0 M0 which was treated with surgery and adjuvant therapy with CT and RT.

In November 2023 she was diagnosed with poorly differentiated gastric signet ring cell adenocarcinoma T3N2M1 with preserved expression of repair genes (pMMR) and was HER2 negative.

#### 2.4.2. Reasons for Consultation

The patient consulted in November 2023 with abdominal pain and lack of stools. An abdominal CT scan showed dilated small intestine loops. She was assessed by the Surgery Department with an exploratory laparotomy that revealed findings compatible with peritoneal carcinomatosis. Given the oncological history of the patient, abdominal, pelvic, and thoracic CT scan and gastroscopy were performed, and the final diagnosis was gastric adenocarcinoma stage IV with peritoneal involvement (IMAGE 1). The analysis showed progressive pancytopenia (platelets 59 × 10^3^/μL, leukocytes 3 × 10^3^/μL, hemoglobin 9 g/dL) and decrease in ADAMTS13 activity (39%). A swab showed presence of schistocytes in the peripheral blood.

The symptoms were classified as TMA secondary to gastric adenocarcinoma. Chemotherapy was administered with a regime of 5-fluorouracyl and oxaliplatin (FOLFOX). The patient showed a poor evolution and died 21 days after hospital admission with multiorgan failure triggered by acute renal failure in the context of TMA.

### 2.5. Case 5

#### 2.5.1. Oncological History

The patient is a 61-year-old woman who had been diagnosed in July 2019 with gastric adenocarcinoma (HER2 negative, NTRK fusion, pMMR, stage cT3N0MX).

Neoadjuvant therapy was administered with 5-fluorouracyl, leucovorin, oxaliplatin, and docetaxel (FLOT), with partial response of the gastric lesion and de novo bone involvement in the PET scan conducted prior to surgery assessment. First line treatment for the metastatic disease was initiated with CAPOX and zoledronic acid which was maintained until December 2022, when the patient presented progression to the gastroesophageal junction as well as axial bone and bilateral scapular involvement. The patient started second line treatment for the metastatic disease with paclitaxel-ramucirumab, which she was still receiving at the time of consultation.

#### 2.5.2. Reasons for Consultation

The patient was admitted as an emergency with minimal–moderate effort dyspnea of 2 months of evolution that had become worse in the last week. Upon arrival, she presented tachypnea (27 breaths per minute), tachycardia (125 bpm), and pressured speech. The analysis presented elevated levels of proBNP (9556 pg/mL) and troponin T (148.4 pg/mL), together with hypoxemia (pO_2_ 59 mmHg), elevated LDH (435 U/L), total Br (2.5 mg/dL), and a decrease in fibrinogen (110 mg/dL) and GF (62 mL/min/1.73 m^2^). An angioCT of pulmonary arteries was performed and it ruled out the presence of pulmonary embolism. A bedside echocardiogram showed severe tricuspid insufficiency and findings compatible with severe pulmonary hypertension (dilation of right ventricle and auricle, PAP 85 mmHg). The patient was hospitalized in the Department of Cardiology for catheter placement and ventilation/perfusion scintigraphy. However, 9 h later, the patient died due to cardiogenic shock. The symptoms were classified as rapidly progressing severe PH secondary to obstruction of microvessels by metastatic emboli in the context of PTTM.

A clinical autopsy was not performed. The case was classified based on the obtained data, which were compatible with PH, and the analytical alterations (decreased levels of fibrinogen, increased bilirubin at the expense of indirect bilirubin, and decreased glomerular filtration) which were compatible with TMA in the clinical context of the patient.

## 3. Discussion

TMA is a complex entity with clinical, analytical, and mainly anatomopathological components [2]. Since schistocytes may or may not be present in peripheral blood, diagnostic certainty requires the biopsy of the damaged organ. However, this is impossible most of the times in the daily clinical practice with patients with an active cancer, given its rapid evolution and the tendency to refrain from conducting aggressive tests if the prognosis is poor and there are few possibilities of remission. Despite the limited availability of organ damage biopsy in routine clinical practice, the early use in the evaluation of oncological patients of classifications such as those proposed by BMT-TCN and IWG, which include clinical–analytical variables, could be useful in the early detection of TMA in patients with active cancer. Although it is true that these scales have been validated in patients with hematological tumors and solid organ transplantation, new studies could explore the proposal of extrapolating their results to patients with solid cancer and TMA, either produced by the tumor itself or as toxicity to treatment.

In our opinion, active-cancer-associated TMA is an underestimated complication which is not generally included in the differential diagnoses in the medical oncology departments and which only receives attention in very advanced stages of the disease, when the symptoms and the analysis alterations are very noticeable and the possibility of achieving remission is virtually inexistent. The extensive bibliography on the prevalence of TMA in patients with adenocarcinomas, especially those of digestive or mammary origin, makes it necessary to emphasize in oncology training programs the existence of this complication and that it should be taken for consideration in oncology patients who begin to show alterations in renal function (as these are the most frequent in TMA) or in other organs and cannot be explained by other causes. In our patient 2, approximately 2 months had passed from the point in which she was assessed after adjuvant therapy and diagnostic tests were requested to the moment that marked clinical deterioration was observed (dyspnea, hypoxemia), which shows the rapid evolution of the symptoms and the narrow margin to make therapeutic decisions. The patient in case 1 had been reassessed the previous month, with findings that showed a stable disease. After observing findings compatible with TMA, an abdominal, pelvic, and thoracic CT scan was conducted that revealed the progression of the disease to the bones and the liver. In this case, we consider TMA to be a manifestation of the advanced disease that involved an invasion of the bone marrow via bone lesions. In patient 3, more than 7 years had passed from the initial diagnosis until the appearance of TMA. As in the case of patient 2, this diagnosis motivated the request for reassessment tests that detected the recurrence of the tumor. The medical literature reported cases of recurrence in gastric adenocarcinomas years after the diagnosis [48]. In spite of adequate monitoring, it was impossible to predict that the patient would develop TMA. We consider that, despite adequate follow-up, it is possible that potentially serious and irreversible complications such as the one suffered by our patient may appear over time. For some medical oncology departments, which are the ones who mainly perform long-term follow-up, it may be unaffordable to evaluate patients with no evidence of disease every 3–4 months, as this would take time away from the care of patients with active cancer and, in many cases, would not provide any clinical benefit. On the other hand, it is audacious and irresponsible to delegate the entire burden of detecting recurrences or potential complications to family physicians, who are the first point of entry into the health care system and, in our setting, the main actors in the follow-up of long-term cancer survivors. In Spain, the agendas of family doctors can in many cases exceed 35 patients per day with pathology variability and limited time for their care. There is a structural problem in which, if we analyze the background, perhaps putting pressure on social agents, politicians, and managers to improve the conditions of family and community medicine would motivate that, with smaller agendas and greater resources, we consider it essential to establish, at least, the evaluation of a protocol that allows the request of preferential imaging tests under appropriate clinical criteria of suspicion or referral/contact on the day with specialists in Internal Medicine and Medical Oncology to discuss relevant cases that require urgent assessment, which is currently limited in many Primary Care Managements. As can be seen, in our opinion, despite the importance of the clinical and pathophysiological mechanisms, major determinants of survival in the case of TMA, the social situation must also be criticized, the improvement of which will always lead to a more rapid diagnosis that allows, in these cases, for the improvement in the prognosis.

Patient number 5 presented symptoms of minimal effort dyspnea of 2 months of evolution when she was assessed. She had been previously assessed up to four times, and she had undergone different tests that did not show noticeable alterations. An echocardiogram revealed very severe pulmonary hypertension. The diagnosis of PTTM requires elevated clinical suspicion, which leads to its being underdiagnosed, according to some authors [15]. In cases such as this patient’s, with a progressive clinical course of two months’ duration, a routine echocardiogram could have been performed as a priority at the onset of the clinical course and, if the presence of PH had been demonstrated, a V-Q scan could have been performed, suspecting that the etiology could be the presence of microemboli. However, it is difficult to know the impact that early diagnostic tests could have on the diagnosis of PTTM. Further work could shed more light on this area.

In advanced stages, as in our case, the prognosis is fatal, and the patient died less than 24 h after she had been admitted as an emergency. The patient was receiving treatment with ramucirumab, a monoclonal antibody that acts against VEGFR2. Some cases have reported kidney toxicity secondary to this drug with the appearance of TMA at this level [49,50]. The medical literature mentions that the appearance of PH secondary to PTTM may be mediated either by VEGF or PDGF [14,17]. This suggests that ramucirumab, a VEGFR2 inhibitor, may be beneficial in the treatment of PTTM, just like imatinib, a TKI that acts against PDGF. The fact that ramucirumab may be effective was mentioned in a case report by Dr Yasuhiro Mitsui [19]. However, the prognosis of this patient was poor, and he died after 17 days. The action mechanism of ramucirumab supports the fact that the mechanism that triggered PH was probably an accumulation of tumor microemboli in a patient with a controlled but active disease.

## 4. Conclusions

TMA in patients with active cancer may be caused by chemotherapy, hematopoietic progenitor transplantation, or the disease itself. Its diagnosis is based on compatible symptoms and on analytical and anatomopathological data. In most cases, a biopsy is not performed for diagnostic certainty, and it is clinical context and experience which make it possible to reach diagnostic suspicion and orient the treatment.

The diagnosis is underestimated. In many oncological patients who present progressive deterioration in the final stages of their disease, the causes are not established, and their worsening condition is attributed exclusively to the progression of the tumor.

An exhaustive monitoring of patients with tumors with a very high risk of complications, such as gastric adenocarcinoma, which includes requesting reassessment tests, control analyses, and detailed anamnesis could lead to an early diagnosis of TMA and to the early implementation of specific treatments. However, the capacities of the National Health System are limited, and a cost-effectiveness analysis is required for any new proposal in this regard.

PTTM is an entity included into TMA. Its diagnosis is very difficult because the usual imaging tests and analytical results in the initial stages may not show any alteration. The experience of the medical professional could motivate requesting specific tests such as ECG or ventilation/perfusion scintigraphy. If an early diagnosis is reached, the combination of treatments against the tumor and drugs such as sunitinib may improve survival in these patients.

Once TMA has been established, the existence of conclusive analytical data and the symptoms lead to a very poor prognosis.

## Figures and Tables

**Table 1:** Triggering clinical pictures of TMA, pathophysiology, and causes.

Table	Physiopathology	Causes
Hemolytic-uremic syndrome (HUS)	Dysregulation of complement and activation of alternative complement pathway	- Typical HUS: siga toxin (*E. coli*) - Infections: Shigella, Yersinia, Klebsiella, Coxackie, HIV - Active cancer - Chemotherapy - Immunosuppressive agents
Thrombotic thrombocytopenic purpura (TTP)	ADAMS 13 deficit	- Congenital - Development of autoantibodies (idiopathic, active cancer, chemotherapy)
Poorly controlled hypertension	Endothelial damage, intimal proliferation, and release of pro-coagulant substances	- Rheumatologic diseases (scleroderma, lupus) - Tumor microemboli - Chemotherapy (platinums, regorafenib) - Radiation
Disseminated intravascular coagulation (DIC)	Excessive thrombin production, phospholipid release, and platelet consumption	- Gram-negative sepsis - Severe trauma - Vascular alterations (Kasaback–Merrit, aneurysms) - Pre-eclampsia and HELLP syndrome - Active cancer - Chemotherapy - Antiphospholipid syndrome

**Table 2:** TMA associated with cancer. Causes, pathophysiology, and treatment.

Type	Causes	Physiopathology	Treatment
Associated with chemotherapy treatment	- Platinum derivatives (oxaliplatin, carboplatin, cisplatin) - Gemcitabine - Mitomycin C - Bevacizumab - Tyrosine kinase inhibitors (TKIs)	- Acute kidney damage, tubular necrosis, injury to renal endothelium - Formation of autoantibodies against erythrocytes and platelets (platinums, gemcitabine) - Phospholipase A2 activation (platinums) - Acute lung damage (mitomycin) - Thrombocytopenia (gemcitabine, mitomycin) - Complement dysregulation	Drug discontinuation Supportive treatment (anti-hypertensives, dialysis, periodic transfusions, corticotherapy) Immunoglobulins Eculizumab (NOT IN INDUCED BY TKIs) Rituximab
Associated with active cancer	- Activation of the coagulation cascade - Dysregulation of the complement system - Vitamin B12 deficiency (gastric fundus neoplasms)	- Direct endothelial damage by tumor microemboli - Megaloblastic anemia and hyperhomocysteinemia that induce red blood cell fragmentation and oxidative stress (in B12 deficiency) - Pro-inflammatory milieu induced by cytokines secreted by tumor cells - Direct activation of the coagulation cascade	Support treatment Tumor-type-specific chemotherapy Imatinib (in some cases of pulmonary TMA)
Associated with hematopoietic progenitor transplantation	- Myelosuppressant-associated toxicity - Immunosuppression (sepsis, bacteremia) - Genetic polymorphisms (SNPs)	- Pro-inflammatory cytokine secretion - Activation of coagulation through cytokines and the complement system - Genetic predisposition - Infections	Plasma exchange (low evidence) Eculizumab Narsoplimab (MASP 2 inhibitor)

**Table 3:** Affectation of different organs with TMA. Target, main, and secondary clinical manifestations, specific analytical alterations, other types of alterations, and results observed in biopsy.

ORGAN	Diana	Main Clinic	Clinic Secondary	Specific Analytical Alterations	Other Alterations	Biopsy
KIDNEY	Glomerular capillaries Glomerulus	Oligoanuria Lower extremity edema Generalized anasarca HTA	Nausea Vomiting Asthenia	Increased creatinine Metabolic acidosis Hyperkalemia Hematuria Proteinuria	Fever Renal fossa pain	Endoluminal thrombi Fibrinoid necrosis Intimal thickening Fibrin thrombi Necrotic endothelial cells C3 tanks Mesangiolysis
LUNG	Arterioles of the pulmonary vasculature Pulmonary capillaries	Progressive dyspnea Asthenia	Orthopnea Weight loss	None Elevation of proBNP Hypoxemia (advanced stages)	EKG S1Q3T3 Tachypnea Tachycardia V/Q defects	Medial and intima-media hypertrophy Increased alveolar macrophages Fibrinoid necrosis Intimal fibrosis
GASTROINTESTINAL TRACT	Intestinal microvasculature	Abdominal pain Diarrhea Ascites	Gastrointestinal bleeding (hematemesis, melena, rectorrhagia)	Increased urea Elevated LDH Indirect bilirubin elevation	Tachypnea Hypotension	Loss of glands Intraluminal schistocytes Intraluminal microthrombi Intraluminal fibrin
CENTRAL NERVOUS SYSTEM	Cerebral microvasculature	Headache Blurred vision Seizures Drowsiness Stupor Hemiparesis Aphasia	Alterations in the visual field Coma	Elevation of LDH CRP elevation	Cerebral edema Ischemic stroke Hemorrhagic stroke PRES Syndrome	Thrombosis in the cerebral microvasculature Intimal hypertrophy in cerebral capillaries (very rare autopsies)

**Table 4:** Clinical and analytical data of the patients included in the sample.

	Patient 1	Patient 2	Patient 3	Patient 4	Patient 5
Gender	Woman	Woman	Woman	Woman	Woman
Age	55–60 years old	75–80 years	70–75 years	65–70 years old	60–65 years
Cancer type	CLI metastatic breast	Metastatic gastric adenocarcinoma	Metastatic gastric adenocarcinoma	Metastatic gastric adenocarcinoma	Gastric adenocarcinoma
Date of diagnosis	April 2021	October 2022	March 2016	November 2023	July 2019
PFS	18 months	7 months	66 months	0 months	5 months
Line of treatment (metastatic)	2L (cisplatin)	1L (paclitaxel)	1L (carboplatin-paclitaxel)	1L (folfox)	2L (paclitaxel-ramucirumab)
GFR (CKD-EPI) mL/min/1.73 m^2^	20	90	89	54	62
Creatinine (mg/dL)	2.56	0.5	0.66	1.2	0.98
Sodium (mmol/L)	137	134	136	130	141
Potassium (mmol/L)	5.2	4.3	4.5	2.7	4.3
ALT (U/L)	105	51	18	67	12
AST (U/L)	82	130	NC	38	NC
GGT (U/L)	121	162	54	61	22
Bilirubin (mg/dL)	2.9	1.1	3.2	2.1	1.2
Direct bilirubin (mg/dL)	1.9	NC	0.9	1.1	0.7
LDH (U/L)	455	1657	2793	309	435
C-reactive protein (mg/dL)	24.8	6.68	5.71	13.81	1.43
NT-proBNP (pg/mL)	172	NC	3243	NC	9556
Troponin T (pg/mL)	3.2	NC	NC	NC	148.4
Hemoglobin (g/dL)	10.9	8.9	8.8	9	12
Leukocytes (×10^3^/μL)	1.52	2.96	4.31	3	12.06
Platelets (×10^3^/μL)	39	24	32	59	274
Reticulocytes (%)	2.4	2.4	13.5	3.5	NC
Reticulocytes (×10^3^/μL)	79	81.5	396.2	98.5	NC
Schistocytes in smears	Present. More than 5%.	Present. Less than 5%.	Present. More than 5%.	Present. Less than 5%.	NC
D-Dimer (mg/L)	2.5	35.1	20.3	NC	2.2
ADAMS-13 activity (normal 40–130–6%)	75%	NC	30	39	NC
TP (ref 7.3–11.8 s)	11.2	13.3	10.3	7.5	11
TTPA (ref 23.2–30.4 s)	37	22.4	30.3	26	24
Fibrinogen (mg/dL) (ref 130–400)	103.1	116.4	234	525.5	110
I.N.R	1.62	1.25	1.24	NC	1.33
Haptoglobin (ref 30–200 mg/dL)			Under 8	NC	NC
Lactate (mmol/L)	3.1	1.4	3.2	3.2	2.5

## Data Availability

The original contributions presented in the study are included in the article, further inquiries can be directed to the corresponding authors.
